# Multi-Criteria Analysis of Startup Investment Alternatives Using the Hierarchy Method

**DOI:** 10.3390/e25050723

**Published:** 2023-04-27

**Authors:** Tamara Kyrylych, Yuriy Povstenko

**Affiliations:** Department of Mathematics and Computer Sciences, Faculty of Science and Technology, Jan Dlugosz University in Czestochowa, al. Armii Krajowej 13/15, 42-200 Czestochowa, Poland

**Keywords:** multi-criteria analysis, criteria composition, investment, startup, Saaty’s method, global priority vector, choosing alternatives

## Abstract

In this paper, we discuss the use of multi-criteria analysis for investment alternatives as a rational, transparent, and systematic approach that reveals the decision-making process during a study of influences and relationships in complex organizational systems. It is shown that this approach considers not only quantitative but also qualitative influences, statistical and individual properties of the object, and expert objective evaluation. We define the criteria for evaluating startup investment prerogatives, which are organized in thematic clusters (types of potential). To compare the investment alternatives, Saaty’s hierarchy method is used. As an example, the analysis of three startups is carried out based on the phase mechanism and Saaty’s analytic hierarchy process to identify investment appeal of startups according to their specific features. As a result, it is possible to diversify the risks of an investor through the allocation of resources between several projects, in accordance with the received vector of global priorities.

## 1. Introduction

The positive tendencies towards economic development require updated business entities according to the current market conditions and the emergence of new structural units, all of which form a competitive economic system. The active development of any economy is not possible without the constant emergence of new economic enterprises. This process stimulates the formation of the market environment with healthy competition and ensures scientific and reproducible functioning. Currently, we observe the positive tendency towards building potential for realizing business ideas through the creation of startups, whose business concepts have been dictated by the needs of the modern society and industries. A startup is a strategic economic unit with innovative concepts with the potential to enter the market. First, we outline the essential features of startups:

(1) the innovation of an idea;
(2) the necessity of capital investment;
(3) reproducibility (possibility to sell the inventive solution multiple times);
(4) business expansion;
(5) the existence of a detailed and structured business plan;
(6) generally, a startup is a project in initial stages of implementation;
(7) the possibility of significant growth of the project;
(8) often, startups propose new technologies;
(9) uniqueness;
(10) the potential team of professionals;
(11) the riskiness of the investments;
(12) the concentration of management decisions by the startup founders;
(13) the flexibility as well as quick and efficient adaptation to changes in the environment;
(14) the possibility to individualize the products, according to the demands of consumers;
(15) the dependence on credit resources;
(16) the close relations between the founder and the employees, etc.

Currently, one of the biggest problems is finding investors for startups, the qualified and objective evaluation of the concepts in terms of costs and benefits for future investment, and the successful presentation of the project to investors. Often, this work is entrusted to consulting agencies that professionally evaluate innovative ideas. For the investor, it is important to have a final estimation containing not only a list of factors justifying the appropriateness of investments in the suggested startups but also the method used for comparing several investment alternatives. For an objective and comprehensive assessment of a startup, a large number of criteria should be taken into account; however, this complicates the evaluation process and prolongs its execution. To assess investment alternatives, many methods, mechanisms, techniques, and tools enable the investigation of investments from different points of view. Research has been concentrated in several directions: the economic basis of startups, the mechanisms of their initiation, and the behaviors of investors. The most substantiated and successful in practice are mathematical models that predict the best investment alternatives. Based on the startup founder’s viewpoint, a comprehensive analysis of investment alternatives should involve the requirements from the idea to launch, from the gathering and successful use of information to the potential of the startup’s innovation in a functioning market. This step-by-step mechanism for building a business was precisely outlined in [1].

The behaviors of investors (especially, business “angels”) towards newly created enterprises in the early stages of their development, the ways of evaluating those enterprises, and the interactions of investors and entrepreneurs were described in [2,3]. The basics of practical venture capital management and the details of the cooperation of venture capitalists and entrepreneurs were presented in [4]. Practical advice and the confirmation of the importance of a correct, accurate assessment of the business opportunities of startups were given in [5]. An analysis of venture capital from the viewpoint of current and future investing in an uncertain environment and the high level of competition confirms complexity of the investment choice [6].

An important step towards identifying the most attractive startup for investment involves not only formulating the list of criteria but also establishing their importance (weights). Today, many consulting companies use expert assignment methods to identify the weights of the criteria, but sometimes, the methods are too subjective and dependent on the composition of the expert team, the expert engagement, and lobbying interests. In this area, special attention is paid to the decision-making theory and the Saaty hierarchy method. The multi-criteria decision-making analysis, known as the analytic hierarchy process, was elaborated by Saaty [7,8,9,10,11,12,13]. This approach has been applied to many areas, such as economics, management, engineering, mathematics, information systems, cybernetics, mechanics, design, chemistry, health service, etc. The literature on this subject is considerable, including the following books [14,15,16,17,18,19] and review articles [20,21,22,23,24,25,26,27,28,29,30,31,32,33], where additional references can be found.

The choice and the comparison of the criteria are important parts of decision-making. As the criteria and their weights can significantly influence decision-making, several approaches to solve this problem have been elaborated. In the analytic hierarchy process (AHP), several prioritization methods have been used for deriving weights, such as the eigenvalue (EV) method [8,10,34,35], the logarithmic least squares (LLS) method [36,37], the weighted least squares (WLS) method [38,39], the fuzzy preference programming (FPP) method [40,41,42,43], and the cosine maximization method (CMM) developed in [44]. A good description of several of the most-used methods was given by Srdjevic [45]. The main feature of the step-wise weight assessment ratio analysis (SWARA) [46,47] is the possibility to estimate the opinions of experts and interested groups according to the significance ratio of the criteria in the process of their weight determination. In the best–worst method (BMW) [48,49,50], two vectors of pair-wise comparison were used to determine the weights of the criteria. The full consistency method (FUCOM) [51,52,53] is based on the pairwise comparison of the criteria and the satisfaction of the mathematical transitivity conditions. The level based weight assessment (LBWA) model [54,55] is suitable for use in complex multi-criteria models with a large number of criteria, and it allows for the additional corrections of the values of the weight coefficients, depending on the preferences of the decision-makers.

The main purpose of this article is to provide a comparison of several startups from the investor viewpoint. In this paper, we discuss the use of a multi-criteria analysis for investment alternatives as a rational, transparent, and systematic approach that reveals the decision-making process during the study of influences and relationships in complex organizational systems. The proposed methods can be useful for consulting agencies, investors, and also for startups founders, who can then assess their competitive position against offers from other competitors in the selected economic branch or industrial sector. The procedure of the startup assessment, especially during the initial stages of implementation (development, operation, execution phases, etc.) is often subjective and challenging, as it requires the determination and account of many indexes as well as extended expert consultation, the formation of criteria, and so on. We propose new criteria and a new criteria composition for evaluating the investment appeal of startups. As an example, we consider three alternative investments in startups: the production of LED traffic lights, the manufacture of information–reference electronic terminals, and the manufacture of rotor-reactive turbo-rotational heaters of liquids. The analysis of the three startups is carried out based on the phase mechanisms and Saaty’s analytic hierarchy process to identify the investment appeal of the startups accounting for their specific features. The consistency index, the consistency ratio, and the global priority vector are calculated. As a result, it is possible to diversify the risks of an investor through the allocation of resources among several projects, in accordance with the calculated vector of global priorities.

## 2. Criteria Composition for Evaluating Investment Attractiveness of Startups

Based upon the review of the literature, the study of the practice of founding and launching startups, successful experiences of investing in startup enterprises, and the results of our previous research, we suggest the following criteria composition, which are consolidated into 12 blocks (Table 1). Similar grouping of sub-criteria into blocks was considered, for example, in [41]. We used several of the block-criteria discussed in [56,57,58,59], and then we supplemented and extended these according to our own criteria.

This criteria could be adjusted according to the economic branch or industrial sector, according to the special features of the business plans presented to the investor. The criteria allow us to analyze the characteristics of startups in a variety of ways, and grouping the proposed criteria could enable potential investors to predetermine the priority groups of the criteria and use the proposed “sketch” of the influential factors to focus attention on the current trends. This criteria-composition model aims to draw the attention of the researcher (investor, consultant) not only on the “classical” list of basic investment indicators (such as payback period and the value of investments) but also to the governmental support of the industry, the innovation and autonomy of startups, time, and resources, as well as the social, scientific, technical, informational, and environmental characteristics.

**Figure 1:**
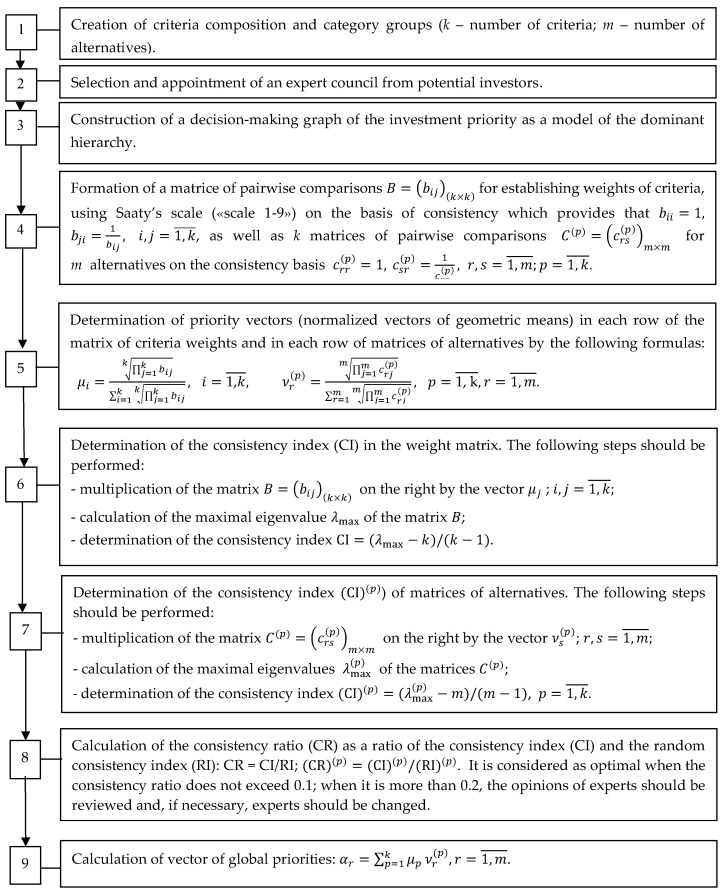
Saaty’s analytic hierarchy process for the identification of the investment appeal of startups based on their specific features.

Figure 1 presents the structure of the Saaty method as the operational algorithm, indicating the priority of investments in startups.

## 3. Implementation of the Saaty Method for Identified Criteria Composition

**Figure 2:**
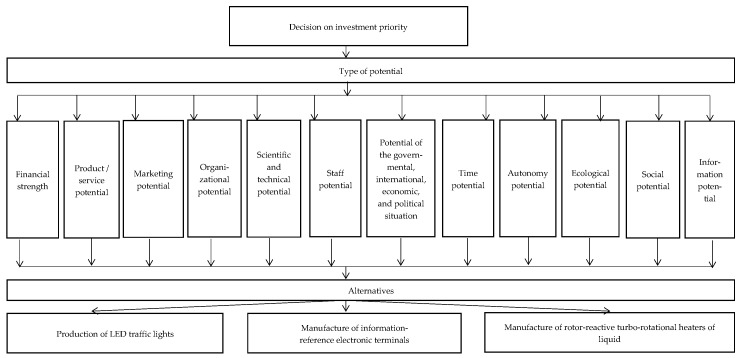
The dominant hierarchical representation of the problem of choosing investment alternatives in startups.

To illustrate practically the Saaty method, we analyze three investment alternatives of startups: the production of LED traffic lights, the manufacture of information–reference electronic terminals, and the manufacture of rotor-reactive turbo-rotational heaters of liquids. The structure of the method is first presented as the dominant hierarchy model in an oriented graph (Figure 2).

After considering the business plans of three investment alternatives and establishing the criteria for assessing the prerogatives of investing in the compared startups, we identified the investment priorities. First, we determined the weights of the criteria according to the sequence of the algorithm; this was the fourth step of the hierarchical procedure, as shown in Figure 1. Table 2 presents the results of the criteria comparison for evaluating the startups using Saaty’s scale (“scale 1–9”) [60,61]. Therefore, we obtained the matrix of pairwise comparisons for establishing the weights of the criteria. The numbers 1–12 in the top row and the first column correspond to the name of the criteria in Table 1. The priority vector ($\mu_{i}$) is calculated as the normalized geometric means in accordance with step 5 (see Figure 1). The column RM presents the results of the multiplication of the paired comparison matrix $B_{ij}$ on the right by the vector $\mu_{j}$. The column DV is obtained by dividing the component of the vector in the column RM by the corresponding component of the vector $\mu_{j}$. The approximation of the maximal eigenvalue is calculated as the arithmetic mean of the components of the vector in the column DV and equals $\lambda_{\max}=12.72$. The consistency index CI=(12.72−12)/11=0.06545. According to [7], for k=12, the random consistency index RI=1.48; therefore, the consistency ratio is CR=CI/RI=0.06545 and does not exceed 0.1.

A similar analysis was performed for the 12 matrices with 3 alternatives. The results for the group of criteria “Financial strength” are shown in Table 3. In this case, we obtain $\lambda_{\max}^{\left(1\right)}=3.0183$. The consistency index ${\left(\mathrm{CI}\right)}^{\left(1\right)}=\left(3.0183-3\right)/2=0.0092$. The random consistency index ${\left(\mathrm{RI}\right)}^{\left(1\right)}=0.52$ for m=3 [7]. The consistency ratio ${\left(\mathrm{CR}\right)}^{\left(1\right)}=0.0158$ and does not exceed 0.1. Taking into account the 12 criteria groups, the final results are shown in Table 4. The conducted research allows us to assert that the startup for manufacturing information–reference electronic terminals is most attractive for investment, as its global priority of 0.3855 is the highest among the analyzed investment proposals. At the same time, the values of the global priorities for the startups producing LED traffic lights and manufacturing rotor-reactive turbo-rotational heaters of liquids are equal to 0.2547 and 0.3599, respectively.

## 4. Concluding Remarks

New criteria and new criteria composition for the comparison of investment alternatives were proposed. Considering the sub-criteria could aid establishing weights of groups of criteria. The criteria and alternatives are mutually independent. A multi-criteria approach based on the analytic hierarchy method was used providing a gradual, clear, and logically structured assessment of the parameters of the given alternatives to ensure a successful solution. The proposed approach also has some limitations. For a large number of criteria and alternatives, Saaty’s scale 1–9 could not be enough. The decision-making process could also be time consuming for a large number of criteria and alternatives. For example, in the case of 32 sub-criteria, there appears a large matrix of pairwise comparisons, and for k>15 in the literature there is no value of the random index (RI) and only an approximate estimation of the consistency ratio (CR) can be obtained. Therefore, we grouped the 32 new sub-criteria proposed in this study into 12 blocks (potentials). Despite these limitations, the AHP approach is one of the most popular and objective methods for multi-criteria decision-making. The proposed use of the Saaty method for optimal decision-making has a number of advantages, as well. It does not require the unification of the units of measurement for different criteria. It ensures the accuracy of the evaluation by increasing the possibility of intra-matching within the selected criteria. In addition, the presence of a numeric scale allows the relations between the factors to be clearly identified. Finally, this method is adaptable, enabling the criteria composition to be modified by adding or eliminating factors. We compared the maximal eigenvalues $\lambda_{\max}$ obtained as the arithmetic mean of the vector in column DV and the value of $\lambda_{\max}$ obtained using the available mathematical package. With a precision of four digits, the results were the same. It should be emphasized that the consistency ratio (CR) of the pairwise comparison of the 12 groups of criteria, as well as all the 12 consistency ratios ${\mathrm{CR}}^{\left(p\right)}$, p=1,2,⋯,12, did not exceed 0.1; therefore, the evaluation was consistent.

In the future, we are planning to extend our research to compare our results with results obtained by other techniques, in particular, using the Bellman–Zadeh fuzzy set approach.

## Figures and Tables

**Table 1:** Criteria composition for evaluating investment appeal of a startup.

No	Type of Potential	Criteria
1.	Financial strength	1. Value of investment 2. Payback period (PBP) 3. Expected profitability 4. Risk level 5. Full or partial investor control of the startup 6. Possibility of reverse repurchase (RRP) 7. Possibility of tranche-funding, depending on the stage of the project
2.	Product/service potential	8. Availability of samples or models of the product
3.	Marketing potential	9. Startup position in the market 10. Forecasted level of demand for the product/service 11. Level of competition in the economic branch or industrial sector 12. Evaluation of startup competitiveness 13. Significant target audience 14. Availability of marketing strategy 15. Requirements to attract and interact with customers within the startup initial stage
4.	Organizational potential	16. Availability of organizational plan
5.	Scientific and technical potential	17. Innovation of idea 18. Innovation of technology 19. Availability of project plan for technical realization 20. Availability of intellectual property rights
6.	Staff potential	21. Availability of potential specialists 22. Uniqueness of specialists
7.	Potential of the governmental, international, economic, and political situation	23. The level of development of economic branch or sector in which the startup will operate 24. The level of governmental support of industry branch
8.	Time potential	25. Period of project completion 26. Stage of project development 27. Duration of product introductory period/start of retail service
9.	Autonomy potential	28. Dependence of the startup on other economic branches or industrial sectors 29. Dependence of the startup on other similar projects
10.	Ecological potential	30. Level of negative impact on the environment
11.	Social potential	31. Accessibility of project’s social utility
12.	Information potential	32. Availability, reliability, and quality of information in economic branch or industrial sector in which the startup will operate

**Table 2:** The matrix of pairwise comparisons to determine the validity of 12 groups of criteria.

Groups of Criteria	1	2	3	4	5	6	7	8	9	10	11	12	$\mu_{i}$	RM	DV
1	**1**	3	3	4	2	1	2	2	4	5	4	3	0.1893	2.3312	12.31
2	1/3	**1**	1/2	1	1/2	1/2	1	1/2	3	3	3	4	0.0800	1.1099	13.87
3	1/3	2	**1**	2	1	1/2	1	1	2	2	2	2	0.0911	1.1345	12.45
4	1/4	1	1/2	**1**	1/2	1/5	1	1/2	1	1	1	1	0.0490	0.6099	12.45
5	1/2	2	1	2	**1**	1	2	1	2	2	2	2	0.1058	1.2968	12.26
6	1	2	2	5	1	**1**	1	2	2	2	2	2	0.1282	1.6571	12.93
7	1/2	1	1	1	1/2	1	**1**	1	1	1	1	1	0.0666	0.8525	12.80
8	1/2	2	1	2	1	1/2	1	**1**	2	2	2	2	0.0942	1.1661	12.38
9	1/4	1/3	1/2	1	1/2	1/2	1	1/2	**1**	1	1	1	0.0483	0.5950	12.32
10	1/5	1/3	1/2	1	1/2	1/2	1	1/2	1	**1**	1/2	1/2	0.0422	0.5329	12.63
11	1/4	1/3	1/2	1	1/2	1	1	1/2	1	2	**1**	1/2	0.0511	0.6742	13.19
12	1/3	1/4	1/2	1	1/2	1/2	1	1/2	1	2	2	**1**	0.0542	0.6975	12.87

**Table 3:** The matrix of pairwise comparisons for the group of criteria “Financial strength”.

Startup	Production of LED Traffic Lights	Manufacture of Information–Reference Electronic Terminals	Manufacture of Rotor-Reactive Turbo-Rotational Heaters of Liquids	Priority Vector (The Normalized Vector of Geometric Means) $\nu_{r}^{\left(1\right)}$	RM	DV
Production of LED traffic lights	**1**	1/3	1	0.20984	0.63337	3.01835
Manufacture of information–reference electronic terminals	3	**1**	2	0.54994	1.65990	3.01833
Manufacture of rotor-reactive turbo-rotational heaters of liquids	1	1/2	**1**	0.24021	0.72503	3.01832

**Table 4:** The optimal choice of startups according to the investment alternatives, based on the groups of criteria.

	Investing Alternatives in Startups	Production of LED Traffic Lights	Manufacture of Information–Reference Electronic Terminals	Manufacture of Rotor-Reactive Turbo-Rotational Heaters of Liquids
**No**	**Groups of Criteria**	**Priority Vectors**		
1.	Financial strength	0.2098	0.5499	0.2402
2.	Product/service potential	0.2000	0.4000	0.4000
3.	Marketing potential	0.2000	0.4000	0.4000
4.	Organizational potential	0.2500	0.5000	0.2500
5.	Scientific and technical potential	0.1634	0.5396	0.2970
6.	Staff potential	0.1958	0.3108	0.4934
7.	Potential of governmental, international,			
	economic, and political situation	0.2500	0.2500	0.5000
8.	Time potential	0.5936	0.1571	0.2493
9.	Autonomy potential	0.1634	0.2970	0.5396
10.	Ecological potential	0.2500	0.5000	0.2500
11.	Social potential	0.3333	0.3333	0.3333
12.	Information potential	0.3325	0.1396	0.5278
13.	Vector of global priorities	**0.2547**	**0.3855**	**0.3599**

## Data Availability

Not applicable.
